# A bibliometric review of hotspots and trends in exosome research over the past decade

**DOI:** 10.1097/MD.0000000000051007

**Published:** 2026-10-09

**Authors:** Tao Zhang, Yujia Han, Yiwei Deng, Xinyue Ren, Dingxing Fan, Xinyu Liu, Haoze Lv, Huanyu Guo, Shilei Lou, Hui Yan, Cong Sun

**Affiliations:** a School of Pharmacy, Changchun University of Chinese Medicine, Changchun, China; b College of Clinical Medicine, Changchun University of Chinese Medicine, Changchun, China.

**Keywords:** bibliometrics, CiteSpace, exosomes, plant exosomes, VOSviewer

## Abstract

**Background:**

This study aims to investigate the recent research hotspots and frontiers in the field of exosomes, offering a comprehensive review of advancements in exosome research and elucidating their implications and future directions.

**Methods:**

English-language exosome-related literature indexed in the Web of Science Core Collection database from January 1, 2015, to December 31, 2024, was retrieved, and the data were analyzed using CiteSpace and VOSviewer software.

**Results:**

A total of 26,458 articles were included. The annual publication volume showed steady growth before 2022, followed by fluctuations and stabilization after 2022. The most prolific author was Takahiro Ochiya; the institution with the highest output was Shanghai Jiao Tong University; China was the leading country in terms of publications and maintained extensive collaborations with other nations.

**Conclusion:**

Current exosome research primarily focuses on pharmacological effects, drug delivery, and diagnostic applications, attracting global scholarly attention and demonstrating promising development momentum.

## 1. Introduction

Exosomes are nanoscale extracellular vesicles, ranging from 30 to 200 nm in diameter, which encapsulate a diverse array of lipids, proteins, and nucleic acids. They are pivotal in mediating intercellular communication, modulating immune responses, and regulating cellular processes such as proliferation, differentiation, and apoptosis.^[1]^ The term “exosomes” was first proposed by Trams et al in 1981.^[2]^ These vesicles were identified in sheep reticulocytes in 1983^[3]^ and formally named in 1987.^[4]^ Exosome biogenesis involves complex steps: invagination of the cell membrane forms early endosomes, which encapsulate nucleic acids, proteins, and other bioactive components. These mature into multivesicular bodies, which fuse with the cell membrane to release exosomes extracellularly.^[5]^ As mediators of cell-to-cell communication,^[6]^ exosomes are pivotal in both physiological and pathological processes.^[7]^ To systematically map the intellectual landscape of this rapidly evolving field, a science mapping approach was utilized to elucidate research hotspots and trends in exosome research over the past decade. This approach was guided by a structured bibliometric methodology.

## 2. Methods

### 2.1. Data sources

A systematic literature search was conducted in the Web of Science Core Collection (WoSCC) using the search query TS=(“exosome” OR “exosomes”) (retrieved on February 28, 2025), spanning from January 1, 2015, to December 31, 2024. Initially, 41,867 articles were identified. Literature screening was conducted by removing duplicate records and excluding nonexperimental studies.

To ensure data integrity, we implemented a 2-step verification process:

• Automatic deduplication: CiteSpace’s duplicate detection function was used to remove duplicate records from WoSCC.
• Manual verification: Two independent researchers (XR and YD) cross-checked 200 randomly selected records to verify compliance with the inclusion/exclusion criteria. Discrepancies (e.g., irrelevant topics) were resolved through consensus or adjudication by a third researcher (HL).

Through the screening process, 26,458 English articles were ultimately selected for further analysis. The analytical procedure is illustrated in Figure 1.

**Figure 1.**
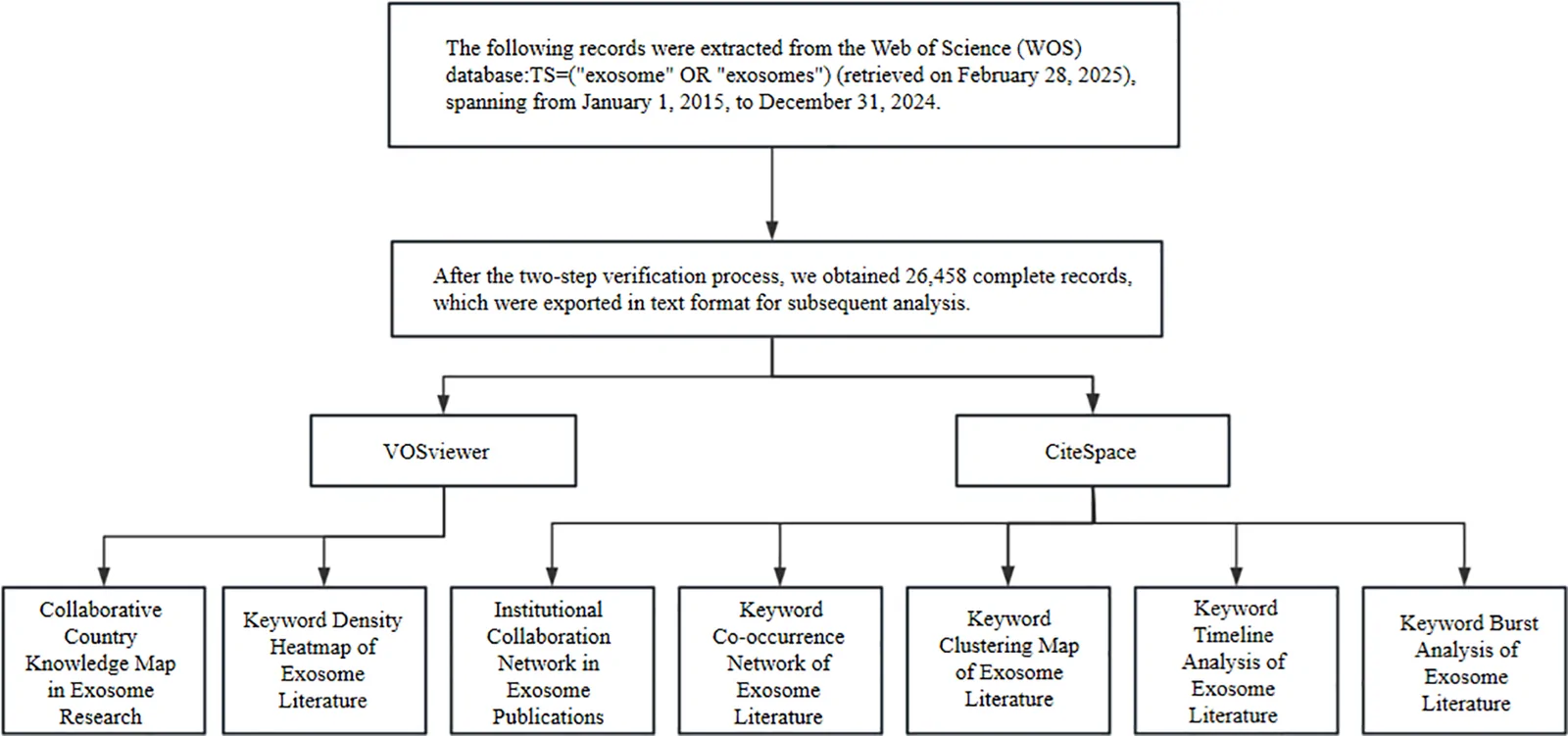
Data retrieval and analysis process.

### 2.2. Data processing

The analysis was performed using 2 specialized bibliometric software tools:

VOSviewer (version 1.6.20; Centre for Science and Technology Studies [CWTS], Leiden University) was employed to generate and visualize the collaborative network among countries and the density of keywords.

The retrieved literature was imported into VOSviewer, and the minimum occurrence threshold for keywords was set to 5. The “Analysis” function was selected from the software toolbar. Parameters across various functional modules were adjusted to ensure a uniform distribution of nodes. Furthermore, to enhance the clarity and aesthetic presentation of the maps, the parameters within the visualization panel were dynamically adjusted. Ultimately, a collaborative country knowledge map and a keyword density heatmap were generated.

CiteSpace (version 6.16; College of Computing and Informatics, Drexel University) was utilized, with data provided in plain text format, to conduct a co-authorship network analysis and to identify keyword bursts, thereby uncovering collaborative patterns and emerging trends.

• Term sources: Title, Abstract, Author Keywords, Keywords Plus.
• Pruning: Pathfinder, Pruning the merged network.
• Visualization: Cluster View-Static, Show Merged Network.

Node density was controlled by adjusting the n-value; other parameters followed default settings.^[8]^

This study adhered to the following bibliometric analysis workflow:

• Network construction: Co-occurrence networks and collaboration networks were constructed based on keyword and institutional nodes, respectively, to elucidate the intellectual structure and research collaboration landscape within the field.
• Thematic clustering and evolution: Clustering algorithms were applied to the keyword co-occurrence network, and a timeline visualization was generated to identify core research themes and trace their diachronic evolution.
• Frontier detection: A burst detection algorithm was executed, with the threshold for the number of burst terms set to 50, to identify and assess emerging research frontiers and development trends in the domain.

The original data used in this study were obtained from publicly accessible databases and did not require ethical review board approval.

## 3. Results

### 3.1. Publication trends

Annual publication trends reflect the discipline’s growth rate, evolution, and maturity. Figure 2 illustrates the annual publication volume of exosome-related research from 2015 to 2024.

**Figure 2.**
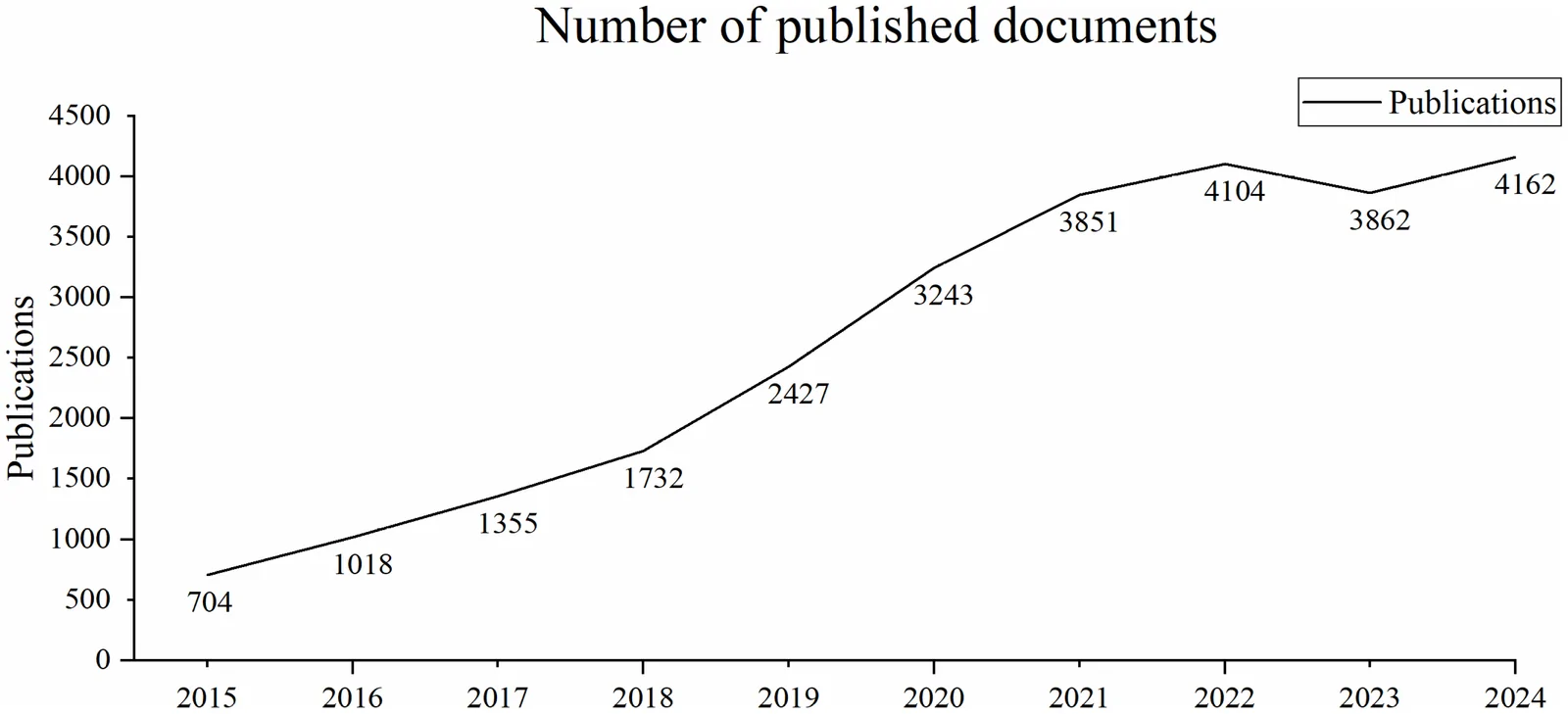
Trends in exosome-related publication volume (2015–2024).

Over the past decade, the number of publications generally increased. Based on annual growth/decline rates, the trend can be divided into 3 distinct phases.

Phase 1 (2015–2018): The publication output on exosomes demonstrated a consistent linear increase, indicative of steady growth in the field.

Phase 2 (2018–2021): A surge in publication volume occurred, reflecting heightened scholarly attention to exosome research.

Phase 3 (2022–2024): The growth rate fluctuated and stabilized, suggesting potential saturation in research interest.

### 3.2. Global research collaboration analysis

A collaborative network visualization of publishing countries was generated using VOSviewer (Fig. 3). In the country collaboration network, larger node sizes indicate higher publication volumes, while denser connections reflect stronger international collaboration.^[9]^ China and the US contributed 13,062 and 5786 publications, respectively, accounting for 71.24% of the total output (Fig. 4). China and the United States lead in exosome research output, demonstrating their substantial influence and extensive collaborations with other nations. Countries currently active in exosome research include China, the United States, Italy, Japan, Germany, South Korea, the United Kingdom, Spain, Australia, and France, with limited representation from African countries. As primary hubs for exosome research, China and the US have accumulated extensive expertise and maintain strong collaborative ties across Asia (e.g., Italy and Japan) and Europe.

**Figure 3.**
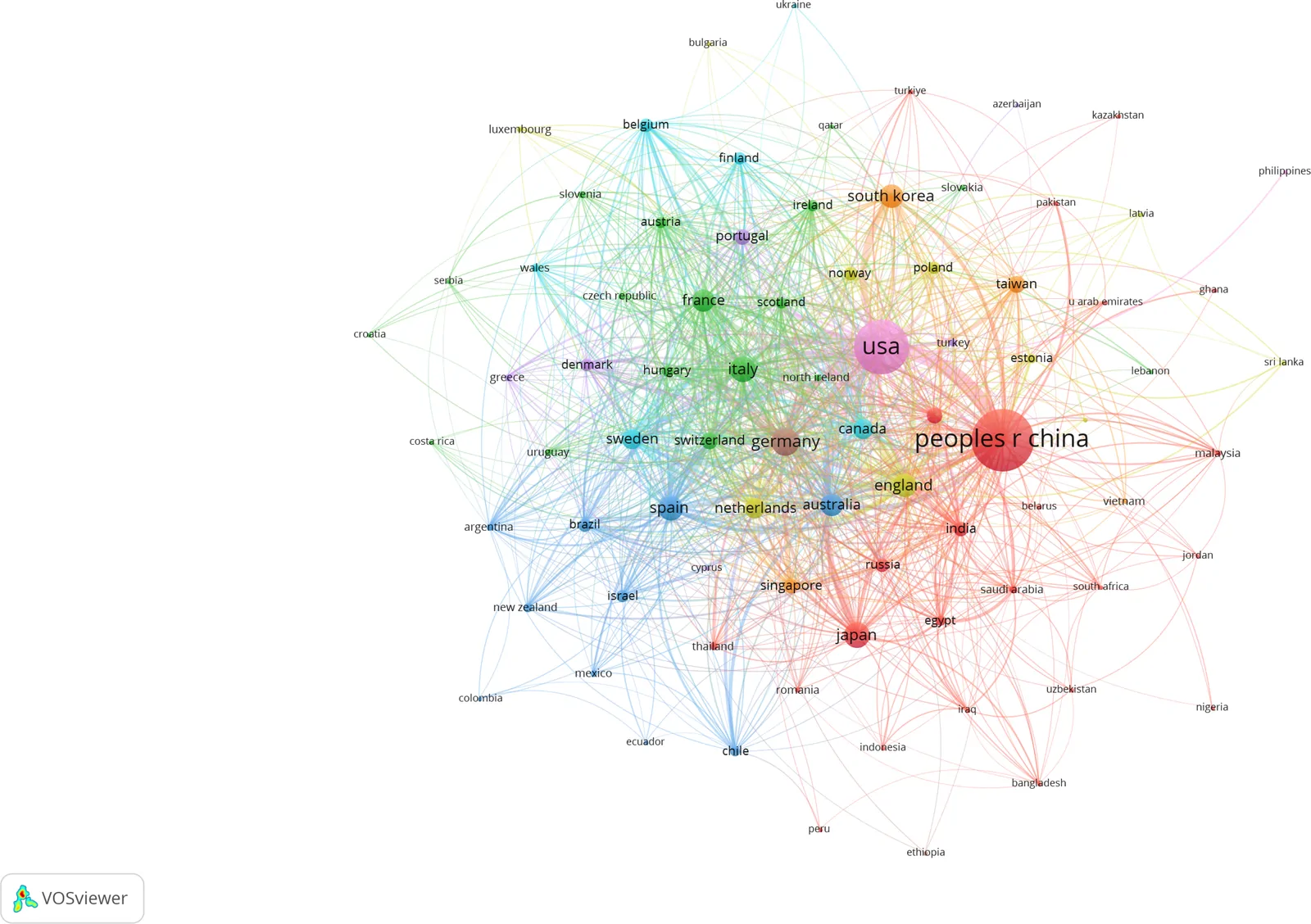
Collaborative country knowledge map in exosome research.

**Figure 4.**
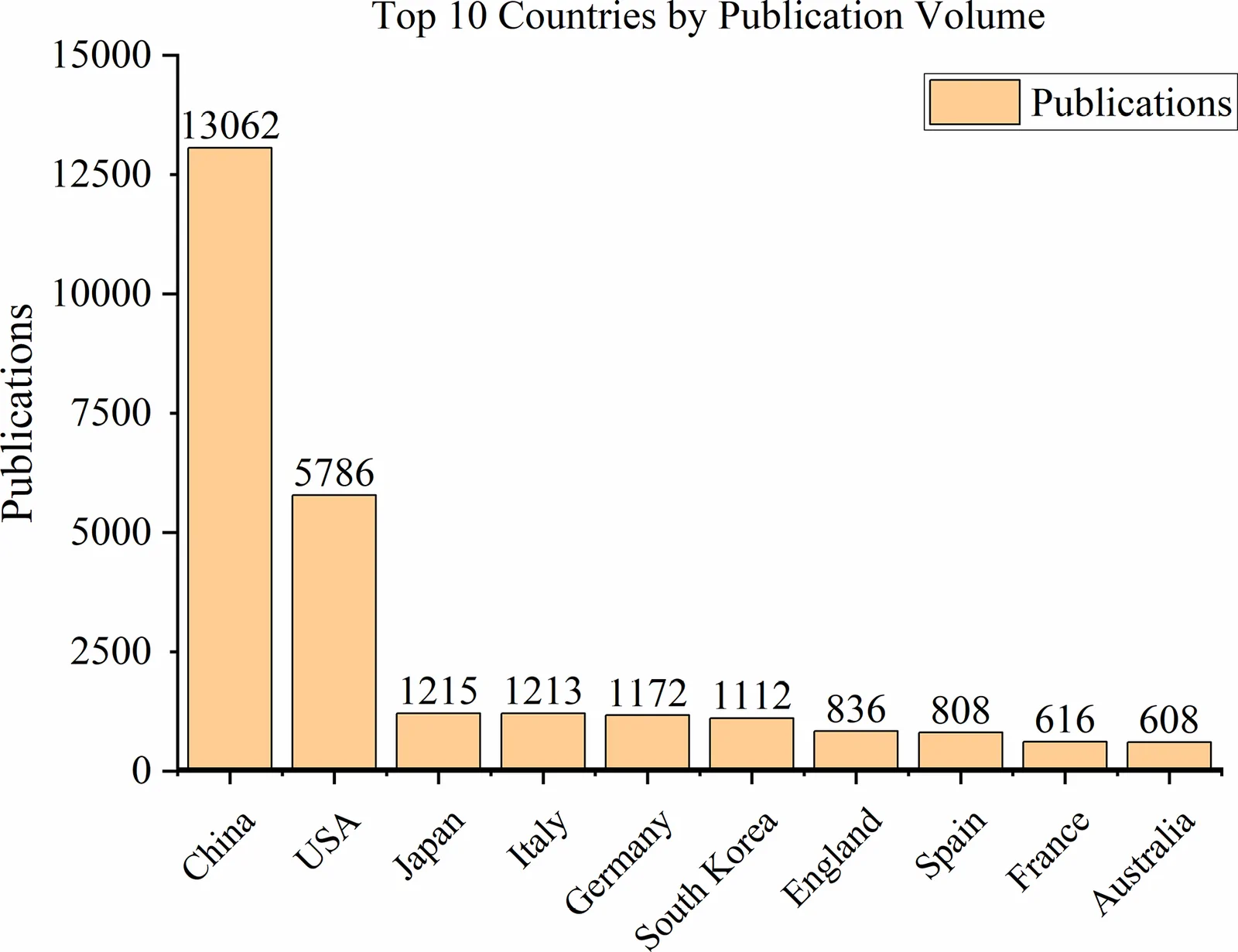
Top 10 countries by publication volume.

### 3.3. Institutional collaboration analysis

A visual analysis of institutional authorship in academic papers was conducted using CiteSpace.^[10]^ The results revealed collaborative relationships among academic institutions, as illustrated in Figure 5, with detailed publication statistics presented in Figure 6. In the institutional collaboration network, nodes represent academic entities (institutions), where node size corresponds to publication frequency or significance. Connections between nodes indicate collaborative relationships, with line thickness reflecting their intensity.^[11]^

**Figure 5.**
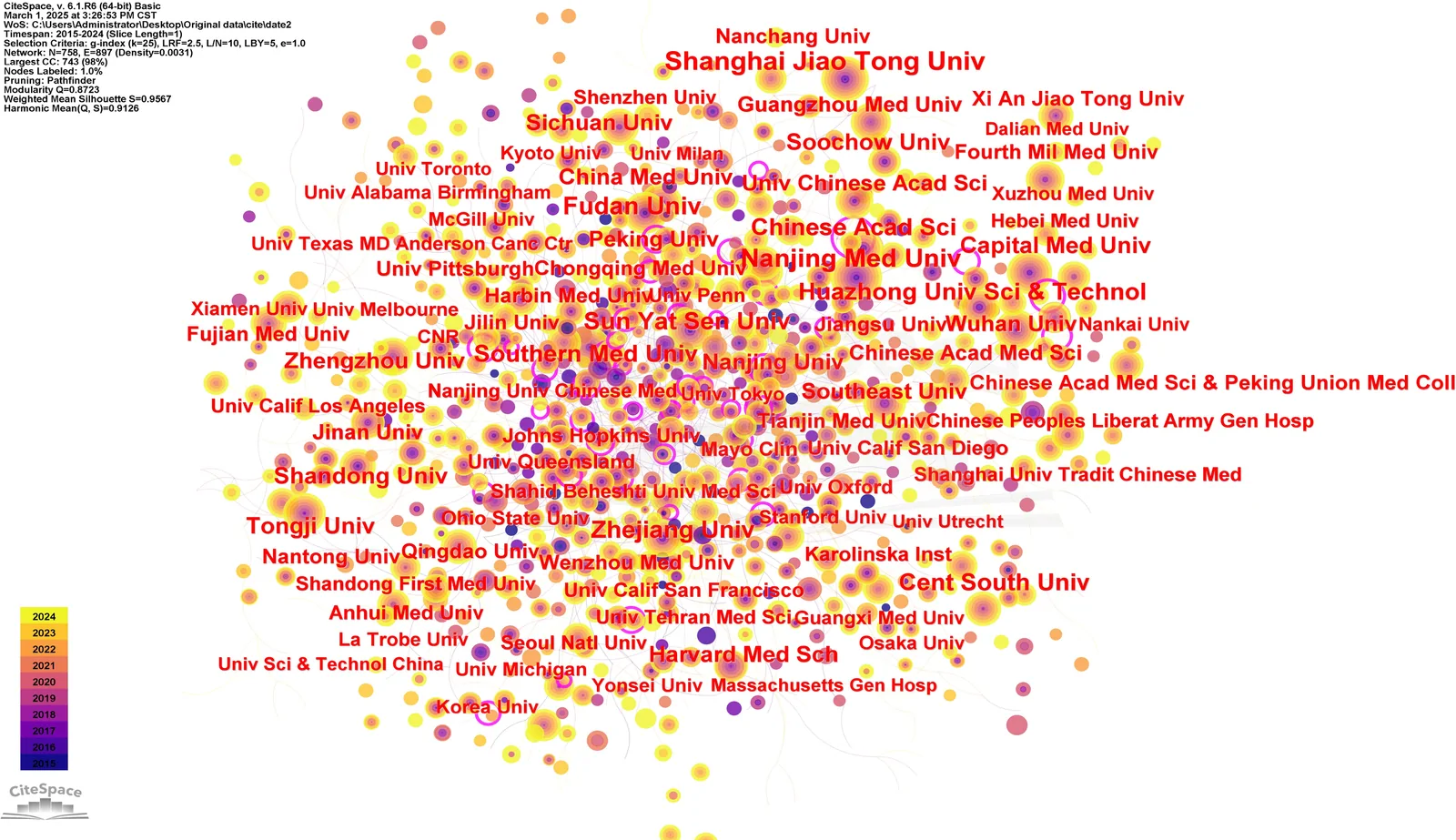
Institutional collaboration network in exosome publications.

**Figure 6.**
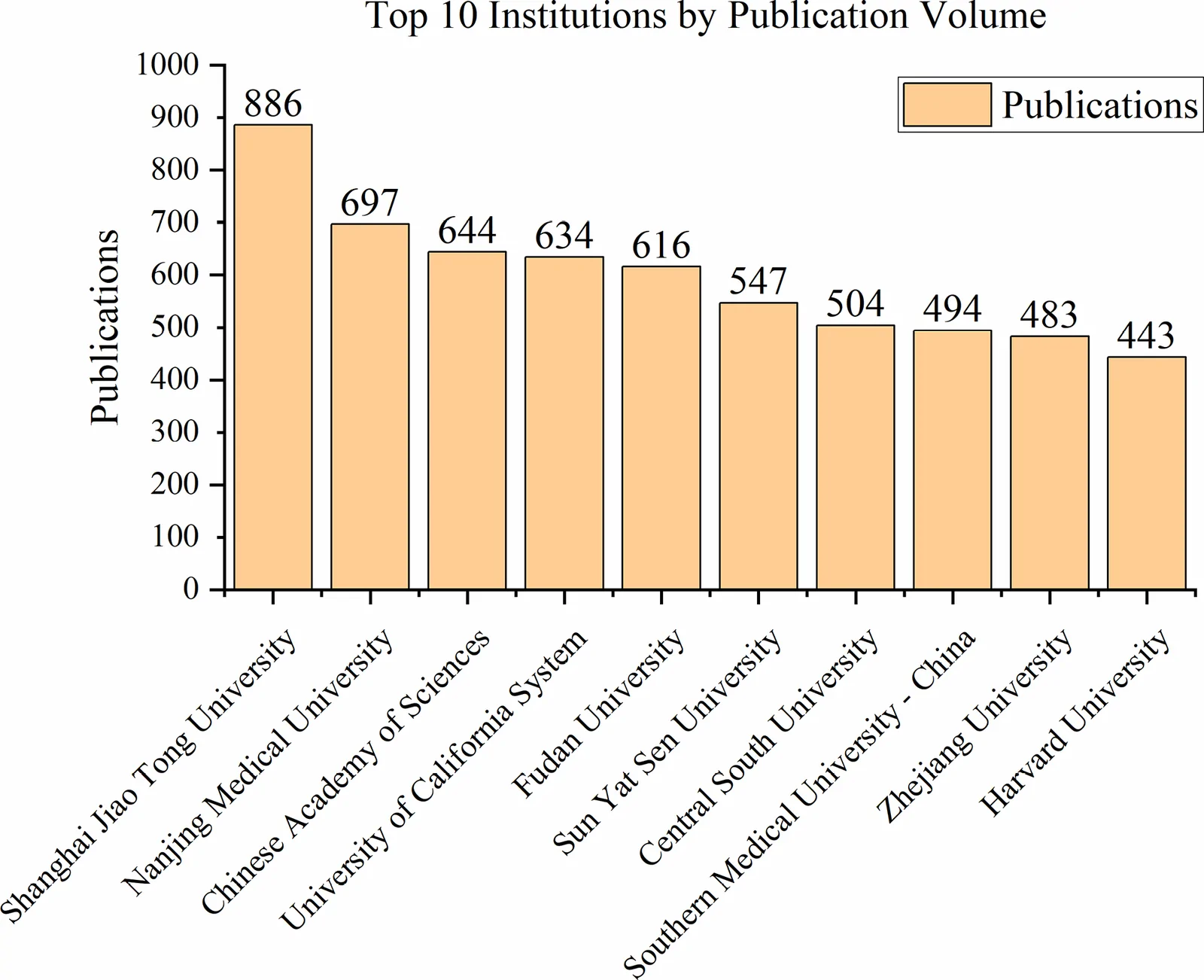
Top 10 institutions by publication volume.

The institutional collaboration network comprises 758 nodes and 5800 links. The top publishing institutions are Shanghai Jiao Tong University (886 publications), Nanjing Medical University (697 publications), Chinese Academy of Sciences (644 publications), University of California System (634 publications), and Fudan University (616 publications).

Notably, 8 of the top 10 publishing institutions are based in China, and 2 are in the United States, which aligns with the national distribution analysis of publications.

Key institutions driving exosome research include the Chinese Academy of Sciences, Jilin University, China Medical University, Southern Medical University, and Shanghai Jiao Tong University, which are primarily medical and comprehensive universities. Collaborative networks are particularly robust between Chinese and US research institutes.

### 3.4. Author analysis

According to Price’s Law, the formula $N=0.749\times \sqrt{\eta }\max$ was used to calculate the publication threshold for core authors, where η_max_ represents the highest number of publications by a single author. Authors with ≥7 publications (rounded up from 6.038) were identified as core authors, totaling 1962 individuals. Details of the top 10 authors are provided in Figure 7. The highest cumulative publication output was attributed to Ochiya, Takahiro (65 publications), a Japanese scholar whose research team has long focused on signaling pathways and biomarkers, achieving significant results.

**Figure 7.**
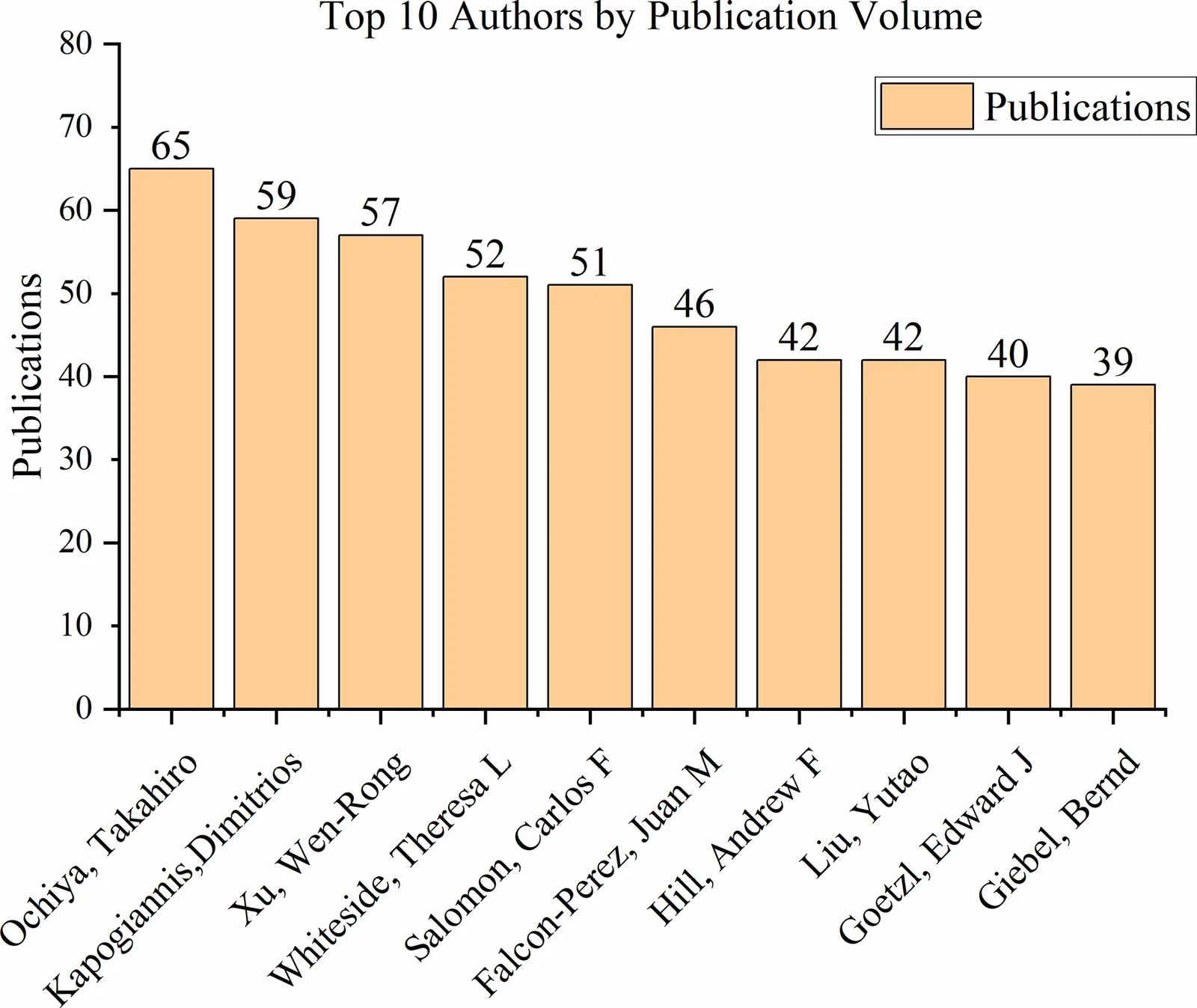
Top 10 authors by publication volume.

### 3.5. Keyword analysis

The keyword co-occurrence network consisted of 918 nodes and 8350 links. Within this structure, 23 keywords emerged with notable prominence, each recurring more than 1000 times, signaling key areas of concentrated scholarly attention. The top 10 keywords predominantly relate to cancer and cellular processes (e.g., apoptosis and autophagy). Keyword co-occurrence mapping (Figs. 8 and 9) revealed concentrated research domains such as tumor biology, cellular mechanisms, and biomacromolecules (e.g., biomarkers). Exosome research has extensively explored their roles in tumor suppression and immune regulation, particularly in cancer-related apoptosis and autophagy. The top 10 high-frequency keywords are listed in Table 1.

**Table 1:** Top 10 high-frequency keywords in exosome research.

Rank	Keyword	Frequency
1	exosomes	7687
2	extracellular vesicle	7577
3	expression	4592
4	cell	3442
5	micro rna	2438
6	protein	2197
7	mechanism	2040
8	cancer	1951
9	microvesicle	1873
10	mesenchymal stem cell	1798

**Figure 8.**
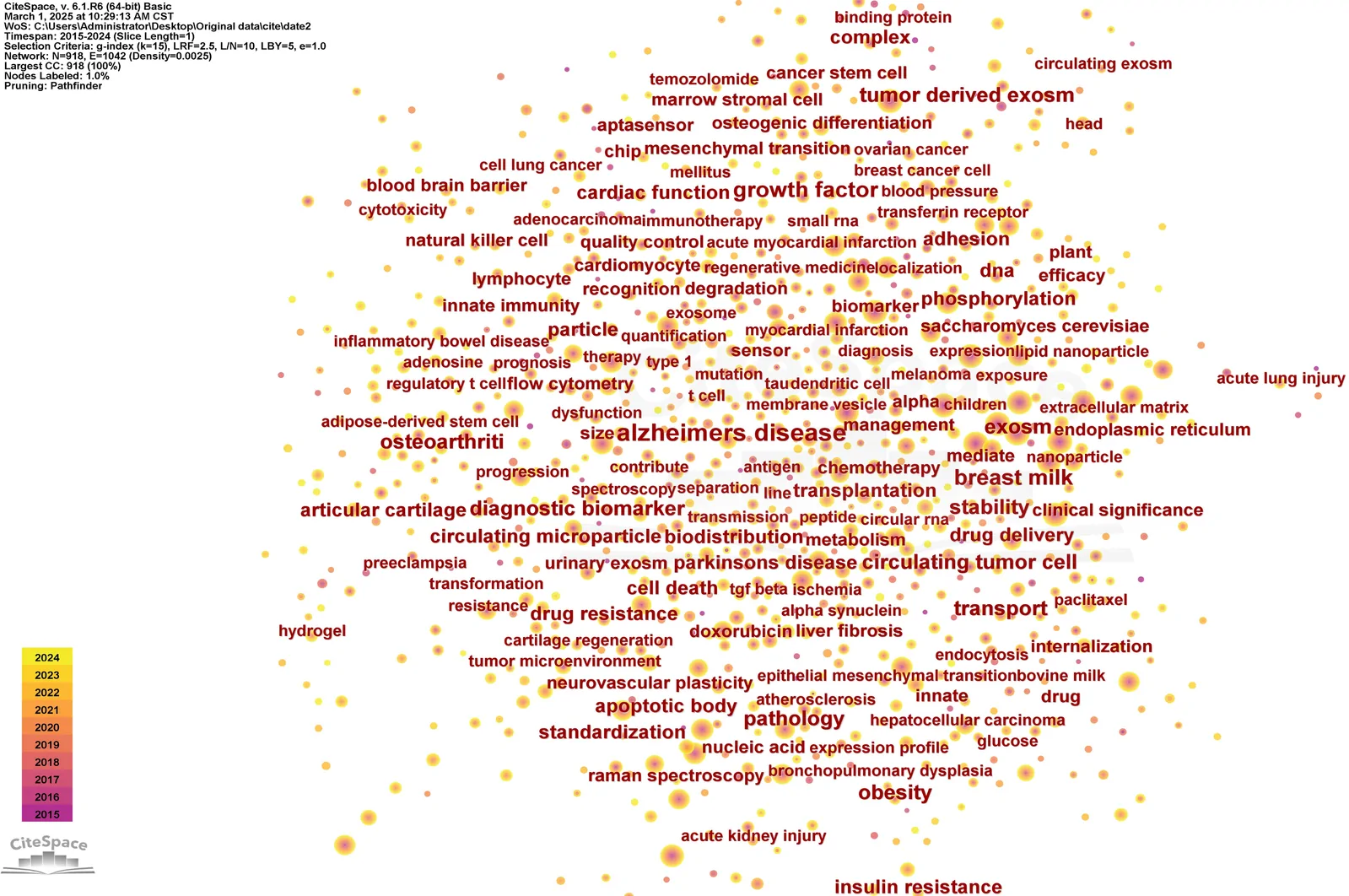
Keyword co-occurrence network of exosome literature.

**Figure 9.**
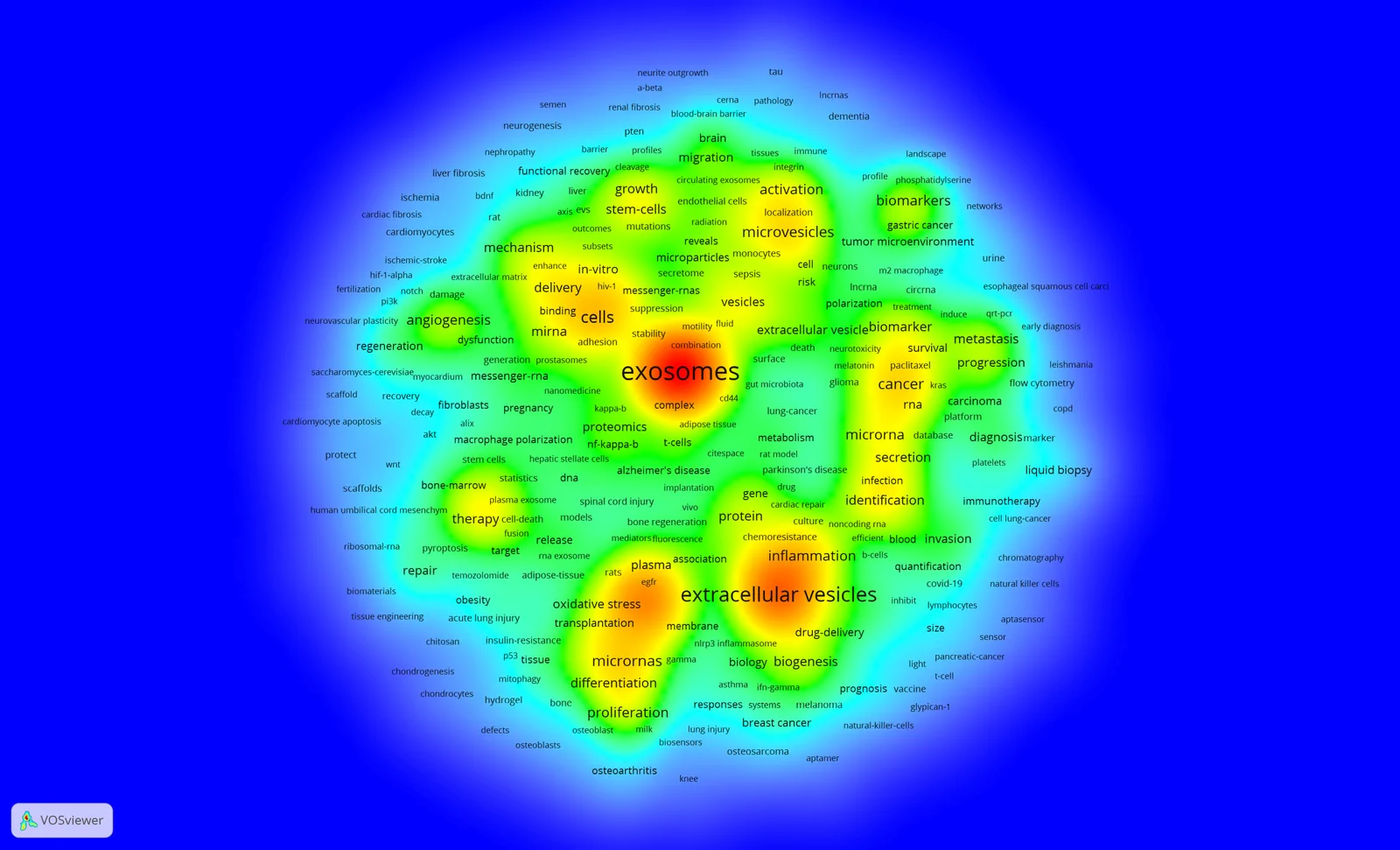
Keyword density heatmap of exosome literature.

### 3.6. Cluster analysis

Keyword clustering analysis plays a pivotal role in bibliometrics by enabling the revelation of disciplinary structures, the identification of research hotspots, and the exploration of scientific knowledge evolution. Cluster analysis of keywords (Fig. 10) generated 26 distinct clusters, and the classification is shown in Table 2. The clustering modularity value (*Q* = 0.8723, >0.3) confirms significant network structure, while the mean silhouette value (*S* = 0.9567, >0.7) indicates high cluster homogeneity and reliability.^[12]^ Clusters #0, #2, #4, #5, #7, #8, #15: Focus on disease mechanisms (e.g., Alzheimer’s disease, lung cancer). Clusters #3, #9, #10, #17, #18, #21, #23, #25: Investigate pharmacological mechanisms of exosomes. Cluster #1: Explores exosome-based drug delivery systems. Clusters #6, #11, #12, #14, #19: Related to cellular studies (e.g., dendritic cells and T cells). Clusters #13, #16, #20, #22: Focus on genetic and transcriptional regulation.

**Table 2:** Cluster information.

Cluster ID	Size	Silhouette	Mean (year)	Label (LLR)
0	51	0.979	2018	alzheimers disease
1	44	0.95	2018	drug delivery
2	44	0.97	2017	breast cancer
3	41	0.902	2017	degradation
4	41	0.946	2019	lung cancer
5	41	0.948	2020	osteoarthritis
6	40	0.939	2017	dendritic cells
7	37	0.972	2018	liver fibrosis
8	37	0.991	2018	pancreatic cancer
9	36	0.978	2019	wound healing
10	35	0.958	2016	activation
11	35	0.994	2019	mesenchymal stromal cells
12	35	0.967	2018	t cell
13	35	0.979	2018	gene expression
14	35	0.953	2018	flow cytometry
15	34	0.939	2017	gut microbiota
16	33	0.951	2018	transcription
17	32	0.892	2017	receptor
18	31	0.977	2016	extracellular vesicles
19	30	0.974	2017	mesenchymal stem cells
20	30	0.96	2015	expression
21	30	0.944	2018	immune response
22	29	0.948	2017	circular RNA
23	29	0.963	2016	protein
24	27	0.935	2017	vesicle
25	26	0.957	2018	osteogenic differentiation

LLR = log-likelihood ratio.

**Figure 10.**
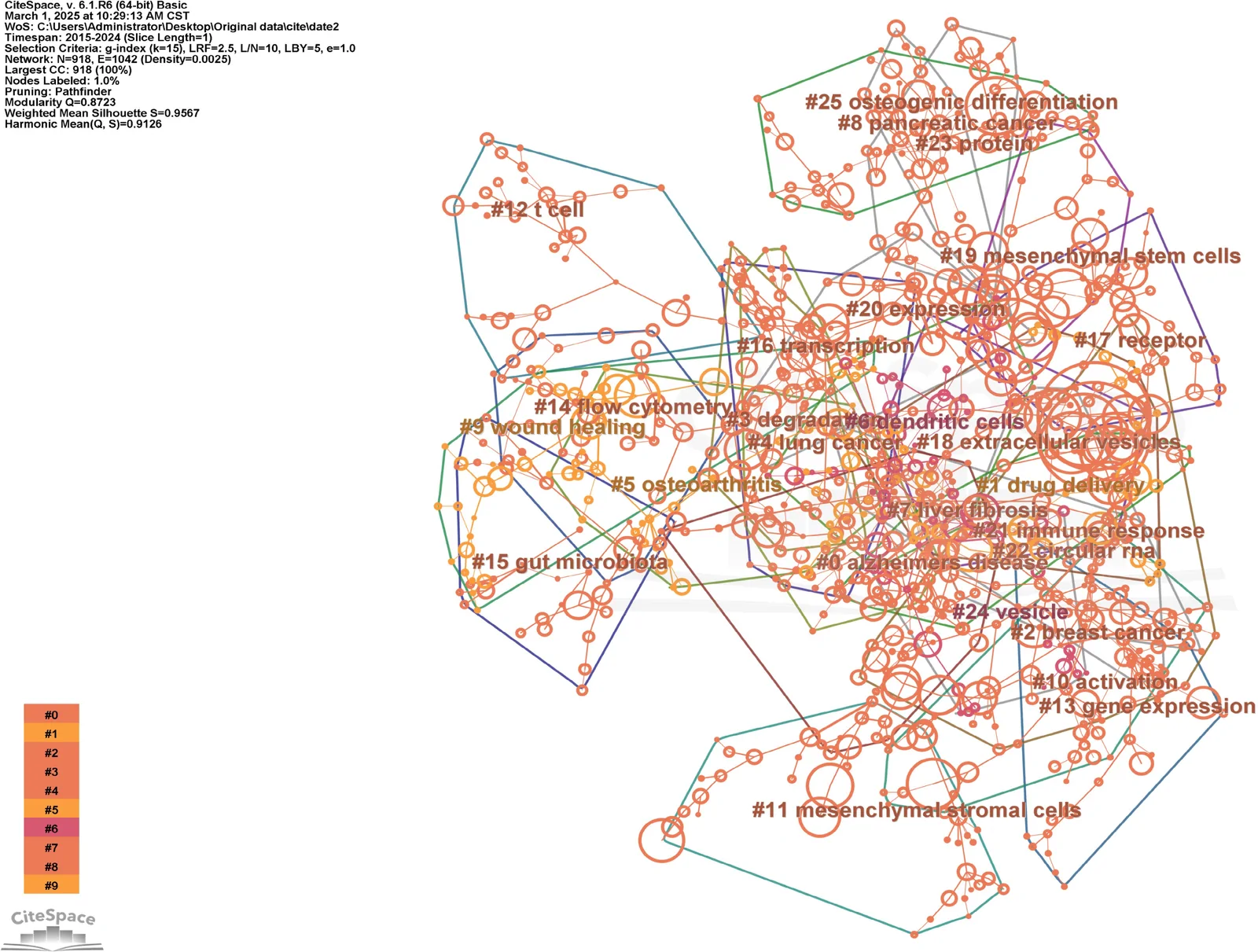
Keyword clustering map of exosome literature.

### 3.7. Timeline analysis

The temporal trajectory of exosome research was investigated through a keyword timeline analysis (Fig. 11). The results show that keywords within each cluster predominantly fall within the 2015 to 2020 timeframe.

**Figure 11.**
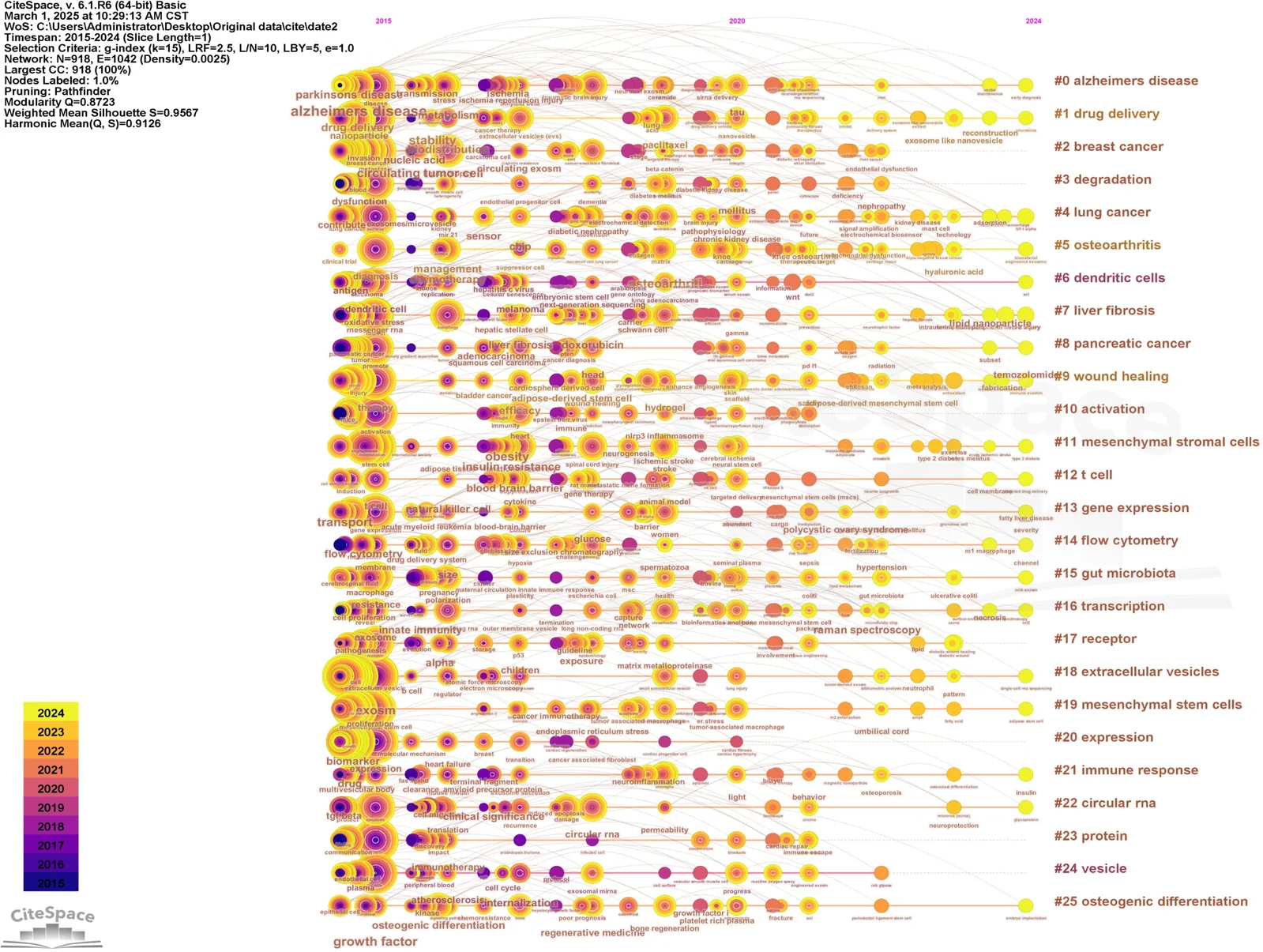
Keyword timeline analysis of exosome literature.

Key clusters with extensive keyword coverage include #0 Alzheimer’s disease, #1 drug delivery, #4 lung cancer, #5 osteoarthritis, #7 liver fibrosis, #9 wound healing, #11 mesenchymal stromal cells, #12 T cell, #14 flow cytometry, #16 transcription, #19 mesenchymal stem cells, and #22 circular RNA. Clusters with timelines extending to the present represent current focal areas. Pharmacological studies on exosomes remain a dominant research frontier.

### 3.8. Burst analysis

The analysis of keyword bursts – terms experiencing a significant surge in frequency over a defined timeframe – was conducted to elucidate emerging trends and pivotal research fronts. The intensity of these bursts was measured by the “Strength” metric. As presented in Figure 12, the application of this burst detection method to exosome research identified 50 keywords, thereby mapping the landscape of historically significant topics and providing a basis for forecasting future trajectories. The highest burst strength was observed for “microvesicle” (127.17, 2015–2018), followed by “membrane vesicle” (54.68, 2015–2017), “circulating microrna” (51.3, 2015–2018), “messenger rna” (49.47, 2015–2018), and “vesicle” (45.29, 2015–2018). Recent bursts (2022–2024) highlight shifts toward “small extracellular vesicles,” “plant,” “gut microbiota,” and “sepsis,” indicating evolving research priorities.

**Figure 12.**
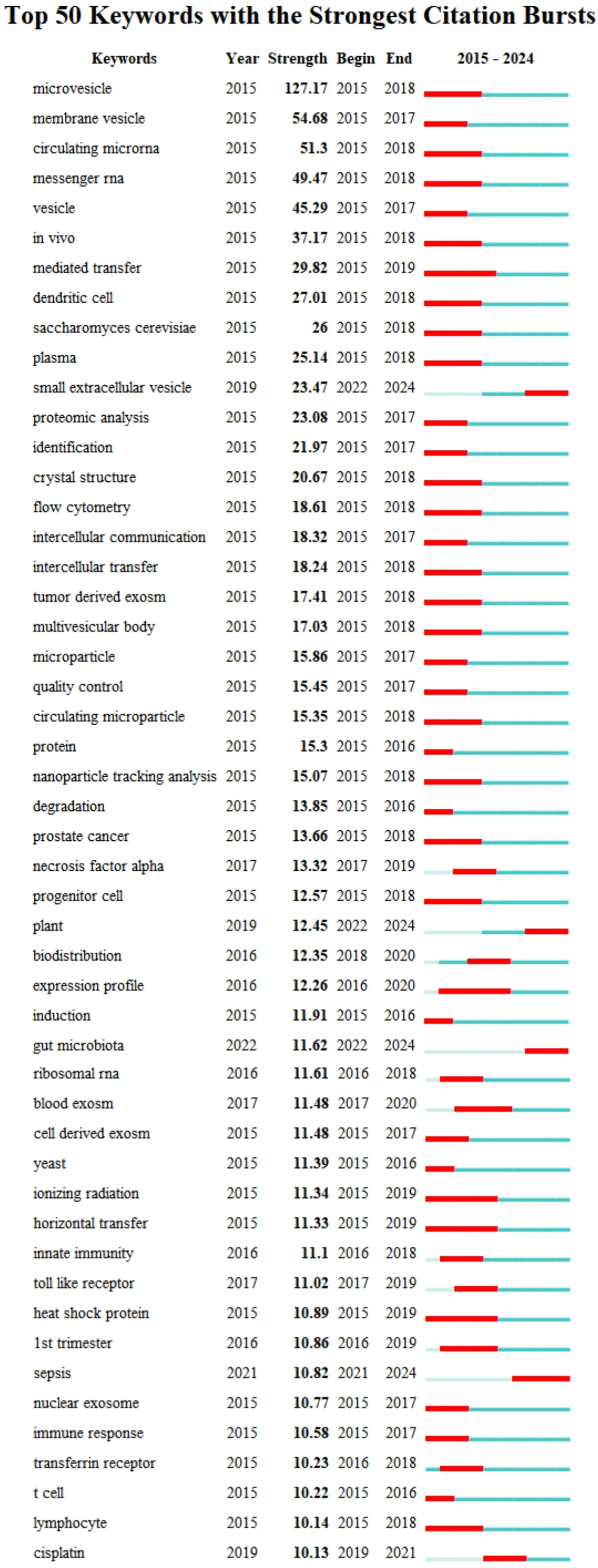
Keyword burst analysis of exosome literature.

## 4. Discussion

Building upon the bibliometric results, this section delineates a detailed analysis structured around the key thematic clusters identified by CiteSpace. As shown in Table 2, keyword co-occurrence clustering yielded 26 distinct clusters with high silhouette values (0.892–0.994), indicating robust clustering quality. The largest clusters encompass Alzheimer’s disease (#0), drug delivery (#1), and breast cancer (#2), reflecting the dominant research themes. The following discussion is organized around these data-driven clusters, covering isolation and characterization techniques, disease implications, therapeutic applications, and emerging frontiers.

### 4.1. Isolation and characterization techniques

Cluster #18 (“extracellular vesicles,” silhouette = 0.977, mean year = 2016) and Cluster #14 (“flow cytometry,” silhouette = 0.953, mean year = 2018) highlight the foundational importance of isolation and characterization methods.

The primary goal of exosome isolation is to obtain high-purity, intact vesicles while avoiding contamination from non-exosomal components (e.g., protein aggregates and lipoproteins).^[13]^ Common methods are summarized in Table 3.^[14–18]^

**Table 3:** Common methods for exosome isolation.

Method	Principle	Advantages	Disadvantages
Ultrafast centrifugation	Separates particles by density and size through sequential centrifugation. Removes cell debris and large vesicles. Ultracentrifugation (≥100,000 × g) pellets exosomes.	No additional reagents. Suitable for large-scale samples (e.g., serum, urine). Considered the “gold standard.”	Time-consuming (6–8 h). Shear force may damage membranes. Poor differentiation from similarly sized particles (e.g., lipoproteins).
Size exclusion chromatography	Uses porous gel matrices to separate particles. Large molecules (e.g., proteins) elute quickly, while exosomes are retained due to smaller size. SEC combined with UC reduces protein aggregate contamination.	Preserves exosome integrity. Avoids high centrifugal force damage. Ideal for small-volume or complex biofluids (e.g., cerebrospinal fluid, plasma).	Limited resolution (co-elution with small vesicles). Low throughput, unsuitable for large-scale processing.
Polymer precipitation method	Adds polymers (e.g., polyethylene glycol) to alter solution hydrophilicity, forcing exosome precipitation.	Simple operation. Cost-effective. No specialized equipment required.	Contamination with non-vesicular components (e.g., protein aggregates). Polymer residues may interfere with downstream analysis.
Immunoaffinity capture method	Utilizes antibodies targeting exosome surface markers (e.g., CD9, CD63, CD81) to capture exosomes via magnetic beads or microfluidic chips.	High specificity. Enriches exosomes from specific sources (e.g., tumor-derived). Compatible with low-volume samples.	High antibody costs. Fails to capture exosomes lacking target markers. Elution may damage membrane integrity.

SEC = size exclusion chromatography, UC = ultrafast centrifugation.

Post-isolation, exosomes require multi-dimensional characterization to confirm purity, morphology, and biomolecular composition. The relatively later mean year of “flow cytometry” (#14, 2018) compared with “extracellular vesicles” (#18, 2016) suggests an evolving trend toward more advanced characterization techniques.

Key techniques include:

Size and concentration analysis^[19]^: Nanoparticle tracking analysis, dynamic light scattering.
Morphological observation^[20]^: Transmission electron microscopy.
Surface marker detection^[21]^: Flow cytometry, Western blot.
Component analysis^[22]^: Proteomics, microRNA (miRNA) sequencing.

### 4.2. Exosomes and diseases

#### 4.2.1. Cancer

Three of the largest clusters in Table 2 are cancer-related: breast cancer (#2, silhouette = 0.970), lung cancer (#4, silhouette = 0.946), and pancreatic cancer (#8, silhouette = 0.991), collectively underscoring the dominance of oncology in exosome research.

Research on tumor-derived exosomes focuses heavily on metastasis and immune evasion. Tumor-derived exosomes carry miR-30a-5p, which modulates DSG2 expression to influence metastatic potential.^[23]^ Tumor-derived exosomes suppress T-cell activity via programmed death-ligand 1 and transforming growth factor-beta signaling.^[24]^ Melanoma stem cell exosomes transfer lncRNA Gm33149 to low-metastatic non-stem cells, promoting aggressive phenotypes via competitive binding with miR-5623-3p.^[25]^

Liquid biopsy techniques utilizing exosomal miRNA profiles or surface protein markers are pivotal for cancer diagnosis.^[26]^

#### 4.2.2. Alzheimer’s disease and Parkinson’s disease

Alzheimer’s disease (AD) constitutes the largest cluster (#0, silhouette = 0.979, mean year = 2018), reflecting its paramount importance in exosome-related disease research. Neuronal exosomes transport β-amyloid, enabling early AD prediction via exosomal Aβ42 detection.^[27]^ Noncoding RNAs (ncRNAs), including long non-coding RNAs, miRNAs, and circRNAs, form complex regulatory networks in AD. This is further supported by Cluster #22 (“circular RNA”), which highlights the growing interest in ncRNA-mediated mechanisms. Exosomes facilitate ncRNA transfer across the blood-brain barrier, offering therapeutic potential.^[28]^

In Parkinson’s disease, α-synuclein toxicity is mitigated by PARK9/ATP13A2 overexpression.^[29]^ Emerging therapies combine Clustered Regularly Interspaced Short Palindromic Repeats/Cas9 with exosome technology.^[30]^

#### 4.2.3. Anti-inflammatory effects

The clustering analysis reveals multiple immune-related clusters – dendritic cells (#6), t cell (#12), and immune response (#21) – collectively indicating that immunomodulation is a central theme in exosome research.

Exosomes exert anti-inflammatory effects through multiple mechanisms, including the direct delivery of anti-cytokine factors. For instance, mesenchymal stem cell-derived exosomes, enriched with interleukin-10 and transforming growth factor-beta, have been shown to effectively inhibit pro-inflammatory cytokines such as tumor necrosis factor-alpha and interleukin-6.^[31]^ Immune cell modulation: Exosomal miR-21-5p promotes M2 macrophage polarization via the SPRY2/ERK axis.^[32]^

Signaling pathway regulation: Exosomal miR-223-3p targets NLRP3, attenuating inflammation through the ASC/Caspase-1/GSDMD pathway.^[33]^

### 4.3. Therapeutic applications

#### 4.3.1. Drug delivery carriers

“Drug delivery” emerges as the second-largest cluster (#1, silhouette = 0.950), confirming its central role in exosome research. Compared with traditional liposomes or synthetic nanoparticles, exosomes exhibit natural targeting ability and enhanced stability, making them ideal drug delivery platforms.^[34]^ Major strategies include chemical drug delivery, nucleic acid delivery (e.g., small interfering RNA and miRNA), and protein/peptide delivery.

Chemotherapeutic drugs can be loaded into exosomes via electroporation or ultrasonication. Tumor-derived exosomes are preferentially internalized by homologous cancer cells,^[35]^ reducing off-target effects on healthy tissues. Exosomes can deliver small interfering RNA to silence oncogenes,^[36]^ supplement tumor-suppressive miRNAs (e.g., miR-34a), and transport Cas9/sgRNA complexes for gene editing. For neurological applications, exosomes loaded with brain-derived neurotrophic factor promote dopaminergic neuron survival in Parkinson disease,^[37]^ while glucagon-like peptide-1 analogs improve insulin resistance in diabetes.^[38]^

Exosome loading methods are categorized as passive loading, active loading, and bioengineered loading.^[39]^ Detailed protocols are summarized in Table 4.

**Table 4:** Exosome loading methods.

Category	Method	Principle
Passive loading	Co-incubation	Drugs enter exosomes via concentration gradients or hydrophobic interactions through simple co-incubation. Relies on physicochemical affinity between drugs and exosome membranes.
Active loading	Electroporation	Transiently disrupts exosome membranes using an electric field to load hydrophilic molecules (e.g., siRNA, mRNA).
	Sonication	Enhances membrane permeability via ultrasonic disruption.
	Freeze-thaw cycles	Facilitates drug encapsulation by disrupting membrane fluidity through repeated freezing and thawing.
	Saponin treatment	Increases loading efficiency by enhancing membrane permeability with saponin.
	Transfection reagents	Mediates nucleic acid binding to exosomes using cationic liposomes.
Bioengineered loading	Overexpression of target proteins	Engineered parental cells to express exosome membrane fusion proteins (e.g., Lamp2b) for targeted delivery.
	CRISPR/Cas9 editing	Engineers exosomes to carry CRISPR systems for gene therapy applications.

CRISPR = Clustered Regularly Interspaced Short Palindromic Repeats, Lamp2b = lysosome-associated membrane protein 2 isoform b, siRNA = small interfering RNA.

#### 4.3.2. Regenerative medicine and immunotherapy

Clusters related to tissue regeneration – including osteoarthritis (#5), wound healing (#9), and osteogenic differentiation (#25) – collectively demonstrate the growing momentum of exosomes in regenerative medicine. Exosomes facilitate tissue regeneration and wound repair by delivering pro-regenerative factors,^[40]^ with applications in cardiovascular repair, neural regeneration, and skin and bone regeneration.

In immunotherapy, exosomes modulate immune cell functions to enhance antitumor responses or suppress excessive inflammation.^[41]^

### 4.4. Plant exosomes

Although plant-derived exosomes do not form a dominant cluster in the current dataset, their absence as a mature cluster paradoxically highlights their status as an emerging frontier. The mean years of most clusters range from 2015 to 2020, suggesting that plant exosome research is still in its infancy within the bibliometric landscape, representing a potential “blue ocean” area.

## 5. Limitations

The core assumption of keyword analysis is “high-frequency words = research hotspots,” and “co-occurring words = thematic relevance.” When keywords are detached from their context and reduced to mere symbols, they fail to reflect the polysemy inherent in specific contexts. Therefore, this implies that keyword clustering solely based on frequency and co-occurrence may identify “hotspots” and “themes” that are merely statistical artifacts rather than profound and accurate reflections of a discipline’s intrinsic structure and dynamic evolution. To avoid reductionist pitfalls, such results require integration with content analysis of literature (e.g., abstracts, introductions, and conclusions).

While bibliometrics holds irreplaceable value in uncovering disciplinary trends and identifying research hotspots, it remains a descriptive tool rather than one for value judgment. It is crucial to avoid equating “what is measurable” with “what is important.”

## 6. Conclusion

This study analyzed 26,458 exosome-related publications (2015–2024) retrieved from the WoSCC using CiteSpace and VOSviewer. Prior to 2022, the publication volume exhibited steady growth, whereas post-2022, it showed fluctuations and stabilized. The most prolific author was Takahiro Ochiya, and the leading institution in terms of publication output was Shanghai Jiao Tong University. China ranks as the top contributor in publication volume and maintains extensive collaborations with other countries.

Current research on exosomes primarily focuses on their pharmacological effects, drug delivery, and diagnostic applications, garnering significant attention from scholars worldwide and demonstrating promising developmental momentum.

However, exosome research still faces several challenges. For instance, achieving standardization remains a critical issue, as exosomes isolated via different methods exhibit significant variations in purity and marker expression, with no current consensus on isolation and quantification protocols. Additionally, scalability poses a barrier due to high costs hindering the translation of research into practical applications. To address these challenges, we encourage research groups to break through existing limitations, foster interdisciplinary, cross-regional, and international collaboration, and strengthen academic exchanges. Such efforts could provide novel insights and directions for future exosome research.

## Author contributions

**Conceptualization:** Tao Zhang, Shilei Lou.

**Data curation:** Tao Zhang, Yujia Han.

**Formal analysis:** Tao Zhang, Yiwei Deng, Xinyue Ren.

**Methodology:** Tao Zhang.

**Resources:** Cong Sun.

**Software:** Tao Zhang, Dingxing Fan, Xinyu Liu.

**Supervision:** Cong Sun.

**Visualization:** Yujia Han, Haoze Lv.

**Funding acquisition:** Huanyu Guo, Hui Yan, Cong Sun.

**Writing – original draft:** Tao Zhang.

**Writing – review & editing:** Shilei Lou.
